# Vessel network extraction and analysis of mouse pulmonary vasculature via X-ray micro-computed tomographic imaging

**DOI:** 10.1371/journal.pcbi.1008930

**Published:** 2021-04-20

**Authors:** Eric A. Chadwick, Takaya Suzuki, Michael G. George, David A. Romero, Cristina Amon, Thomas K. Waddell, Golnaz Karoubi, Aimy Bazylak

**Affiliations:** 1 Thermofluids for Energy and Advanced Material Laboratory, Department of Mechanical and Industrial Engineering, Faculty of Applied Science and Engineering, University of Toronto, Toronto, Ontario, Canada; 2 Latner Thoracic Surgery Research Laboratories, University Health Network, Princess Margaret Cancer Research Tower, Toronto, Ontario, Canada; 3 Advanced Thermal/Fluid Optimization, Modelling, and Simulation (ATOMS) Laboratory, Department of Mechanical and Industrial Engineering, Institute of Biomedical Engineering, Faculty of Applied Science and Engineering, University of Toronto, Toronto, Ontario, Canada; Charite Universitatsmedizin Berlin, GERMANY

## Abstract

In this work, non-invasive high-spatial resolution three-dimensional (3D) X-ray micro-computed tomography (μCT) of healthy mouse lung vasculature is performed. Methodologies are presented for filtering, segmenting, and skeletonizing the collected 3D images. Novel methods for the removal of spurious branch artefacts from the skeletonized 3D image are introduced, and these novel methods involve a combination of distance transform gradients, diameter-length ratios, and the fast marching method (FMM). These new techniques of spurious branch removal result in the consistent removal of spurious branches without compromising the connectivity of the pulmonary circuit. Analysis of the filtered, skeletonized, and segmented 3D images is performed using a newly developed Vessel Network Extraction algorithm to fully characterize the morphology of the mouse pulmonary circuit. The removal of spurious branches from the skeletonized image results in an accurate representation of the pulmonary circuit with significantly less variability in vessel diameter and vessel length in each generation. The branching morphology of a full pulmonary circuit is characterized by the mean diameter per generation and number of vessels per generation. The methods presented in this paper lead to a significant improvement in the characterization of 3D vasculature imaging, allow for automatic separation of arteries and veins, and for the characterization of generations containing capillaries and intrapulmonary arteriovenous anastomoses (IPAVA).

## Author summary

Chronic pulmonary diseases are a leading cause of death responsible for 4 million deaths per year worldwide. Pulmonary diseases attack both the airways and vasculature of the lungs, restricting the oxidation of blood delivered to the rest of the body. For studying disease treatments, the vasculature is of particular importance as it is used as the pathway for drugs as well as nutrients for the case of regenerative medicine. Imaging techniques can be effective tools for studying the effectiveness of such treatments by providing a three-dimensional map of the vasculature before, during, and after treatment. Lung vasculature is incredibly complex and therefore requires great care in the imaging acquisition and analysis phases. Therefore, we have developed a detailed methodology for extracting the vasculature as a network of connected cylinders with accurately estimated measurements and connectivity of the entire vasculature network. The network can be used for simplified models of drug delivery or as a way of benchmarking the topology of a diseased, damaged, or regenerated lung against a healthy lung. The methods presented are not necessarily specific to lung vasculature and may also be applied to images of airways, tree roots, or other similar structures.

## Introduction

Globally, chronic pulmonary diseases are responsible for four million deaths per year [1]. Quantifying the changes in lung morphology is highly desirable to further the understanding of pulmonary disease and develop disease treatments. For quantitative measurements of aberrant morphology, X-ray computed tomography (CT) has been used in the study of pulmonary vascular disease [2–5], vasculature quantification and fluid simulations in tumors [6–9], and tissue engineering methods such as de- and recellularization of whole organs including the lungs [10]. X-ray computed tomography is an imaging technique that provides high-resolution 3D images with resolutions ranging from several millimeters down to several micrometers via micro-computed tomography (μCT). X-ray μCT is an attractive method for studies of pulmonary vasculature due to the high-spatial resolution available (typically from 50 μm to 2 μm per voxel) that allows for the visualization of higher generations of the vasculature and even capillary structures.

The use of high-density contrast agents in μCT imaging has been successful for improving contrast, reducing scan times, and confidently resolving higher generations in airways and pulmonary vasculature of small rodents [4,10–15]. However, the 3D data created by μCT analysis contains continuous grayscale information for which each data point is correlated to the local density of the sample. In order to analyze this data, image processing techniques must be applied to quantitatively segment specific structures of interest. Binary segmentation is the most straightforward approach to analyze this data; however, more sophisticated methods have been developed for the identification of vessel-like structures [16,17]. These methods of segmentation allow for identification of the vasculature but can experience limitations when used across a broad range of vessel sizes, are sensitive to signal noise in the data, and require further processing to achieve quantitative analysis.

Various image analysis methods have subsequently been used to quantify the pulmonary vasculature in terms of the physical characteristics of the vessels (e.g. diameter and length) with subsequent classification of vessels into generations [4,11,12]. Characterization methods typically employ a centreline extraction or skeletonization of the segmented μCT images [4,11,12,18–27]. During the skeletonization step, erroneous centrelines known as spurious branches are bound to appear due to signal noise on the boundaries of vessels in the 3D images. Since spurious branches are often much smaller in length compared to their connecting vessel, the undetected presence of spurious branches may lead to confusion when attempting to identify microvasculature, such as capillaries. This complication is a significant source of error in the quantification of vascular morphology [20,22–25]. Other researchers have presented methods for removing spurious branches in vasculature imaged with voxel sizes ≥ 20.70 μm [20–24]. However, with lower voxel sizes (i.e. higher spatial resolution) and the subsequent detection of smaller vessels (e.g. capillaries), the use of previous methods of spurious branch removal may lead to the erroneous removal of small vessels. Thus, there is a critical need for a robust and consistent approach to identify and remove spurious branches observed in high-resolution μCT imaging of lung vasculature.

Capillaries play a key role in gas exchange. Gas exchange occurs in the capillaries where deoxygenated blood entering from the arteries exchanges carbon dioxide for oxygen before flowing out of the capillary bed and into the veins. Therefore, μCT imaging provides a valuable means to evaluate diseased or damaged pulmonary circuits by capturing and quantifying the capillaries and identifying the arterial and venous sides of the pulmonary circuit. Due in part to constraints on imaging resolution [4], capillaries for mouse pulmonary circuits were not identified in earlier studies. Counter *et al*. [4] distinguished the arterial side from the venous side of the pulmonary circuit with a voxel size of 115 μm. However, due to this relatively large voxel size, the expected connecting capillaries between the two sides were not resolved. Phillips *et al*. [11] captured and characterized vessels with diameters up to 25 μm with a lower voxel size of 8 μm. However, capillary transit was purposely prohibited in [11] by using a contrast agent with a high viscosity. Vasquez *et al*. [13] detected vessels down to 30 μm in diameter with a voxel size of 10 μm by using a contrast agent with a lower viscosity to reach smaller vessels; however, complete quantification was not a goal of their study and thus they did not identify capillaries. To the best of our knowledge, the entire mouse pulmonary circuit (including the capillaries) has yet to be quantified. Furthermore, at lower voxel sizes of ≤10 μm, studies have not used the connectivity of the vascular tree to differentiate between the arterial and venous sides of the pulmonary circuit. Examining these connections will lead to a robust classification of capillaries and thereby a greater understanding of the entire pulmonary circuit for studying diseases, vascular development, and tissue engineering.

Premature connections between the arterial and venous sides of the pulmonary circuit (prior to the capillary bed) have been considered as artefacts (referred to as “loops”) resulting from image reconstruction and analysis in previous μCT studies [11,24,25]. Phillips *et al*. [11] reported errors while using commercial software for classifying generations if loops are present. Whereas loops may indeed be caused by reconstruction noise, intrapulmonary arteriovenous anastomoses (IPAVA) also manifest as connections prior to the capillary bed. IPAVA are small passages that prematurely connect the arterial and venous sides of the pulmonary circuit [28]. Previous studies have identified shunts qualitatively (visually) in reconstructed radiography images [28,29]; however, the quantitative identification of IPAVA in 3D has not been reported in literature.

In this study, we propose a novel Vessel Network Extraction algorithm to improve centreline accuracies via automated removal of spurious branches. We then use the improved centrelines to quantify key physical characteristics of the vessels such as length, diameter, and connectivity. The μCT images in this study are acquired at a sufficiently high spatial resolution and field of view to capture the entire pulmonary circuit including microvasculature. Additionally, we present a novel method of classifying vessels in the pulmonary circuit as arterial, connecting, and venous vessels based on vessel connectivity. Connecting vessels were considered either IPAVA or capillaries depending on the generation they resided in.

## Methods and materials

All algorithms presented in this methodology were developed in MATALB R2018a (MathWorks, Natick, MA, USA). External codes implemented in this work are listed in S1 Table. To characterize the branching morphology of mouse pulmonary circuits we first performed high-spatial resolution three-dimensional (3D) X-ray imaging on mouse lungs. A single set of mouse lungs are presented in this study.

The X-ray images were then filtered, segmented, and skeletonized to elucidate the full pulmonary circuit. Due to noise at the vessel boundaries, artefacts known as spurious branches (non-physical) were observed in the skeletonized image. These imaging artefacts were eliminated with the following three steps. First, spurious branches were removed via a *gradient filter*, to remove branches that approach vessel boundaries. Second, spurious branches were removed via a *diameter-length filter*. Finally, spurious branches were also removed via an *faster marching method (FMM) filter*. The remaining centrelines (herein referred to as *vessel centrelines*) in the skeletonized image were characterized to determine their lengths, diameters, and connectivity. Lastly, we characterized the branching morphology of the pulmonary circuit by determining the mean vessel diameter of each generation, the number of vessels that appeared in each generation, and the connectivity linking the arterial and venous sides of the circuit. Total processing time including filtering, segmentation, skeletonization, and the vessel network extraction was approximately 150 hours for a 16GB 8-bit stack of images. Computing was performed on a computer with 128GB of memory, 16 cores, and a processor speed of 3.75Ghz.

### Imaging of mouse lungs

**Fig 1:**
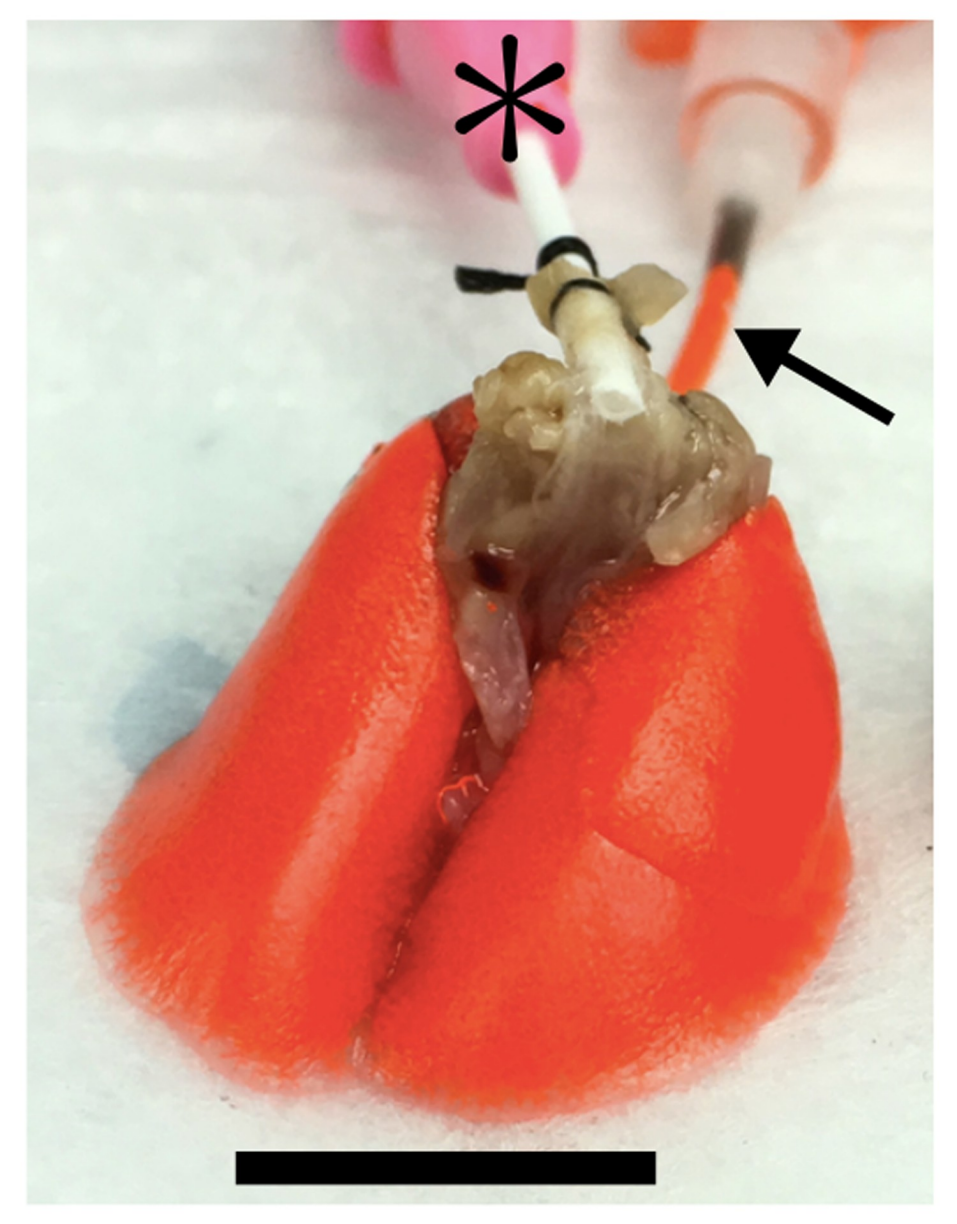
A set of mouse lungs filled with liquid contrast agent. Contrast agent (orange) was injected through the pulmonary artery (black arrow). The second cannulation pictured (*) was for the trachea and was not used in this study. Scale bar represents 10mm.

All animal care protocols and procedures were performed in accordance with relevant guidelines and with approval by the Institutional Animal Care and Use Committee of the University Health Network (Toronto, Ontario, Canada). Following euthanasia, 12-week old adult C57B1/6 mouse lungs were exsanguinated and injected via the pulmonary artery with a liquid contrast agent (Microfil MV-117, Flow Tech Inc., Carver, MA, USA) to enhance vessel intensities during X-ray imaging. The contrast agent was prepared at 8:10:1 parts contrast agent-to-diluent-to-curing agent to form a solid cast of the pulmonary circuit. The injection of the contrast agent was performed via the pulmonary artery at a flow rate of 0.1 mL/min using a syringe pump (NE-1000, New Era Pump Systems Inc.). The whole lung sample used in this study was injected with the contrast agent under non-inflated conditions. A non-inflated lung was used because it has been proposed that non-inflated lungs may enhance peripheral circulation (circulation to smaller blood vessels) while inflated lungs may enhance central circulation (circulation to larger or major blood vessels) [30]. Lungs filled with the liquid contrast agent are shown in Fig 1. Following the injection of the contrast agent, the mice were incubated for 18 hours at 4°C to allow the contrast agent to cure and form a solid cast of the vasculature. The lungs and heart were then surgically removed from the thoracic cavity and fixed in 4.0 wt. % paraformaldehyde for 24 hours to prevent tissue decay from autolysis and putrefaction.

#### X-ray μCT imaging of mouse lungs

To acquire 3D images of the lungs, the lung was first wrapped in ethanol-soaked cotton and placed in a test tube. The test tube was then fixed to the mounting stage and imaged using an X-ray micro-computed tomography system (SkyScan 1172, Bruker Corporation) with a 0.5 aluminum filter at a voltage of 60 kV, a current of 168 μA, a rotational step of 0.25°, exposure time of 4.123 s, and averaging of 4 frames. Ethanol was used to ensure sample hydration, mitigate shrinkage, and prevent bacterial contamination. The cotton was used to support the mounted samples and prevent sample movement during imaging. The pulmonary circuit was captured in the lungs at a voxel resolution of 4.97 μm (spatial resolution of 14.91 μm).

**Fig 2:**
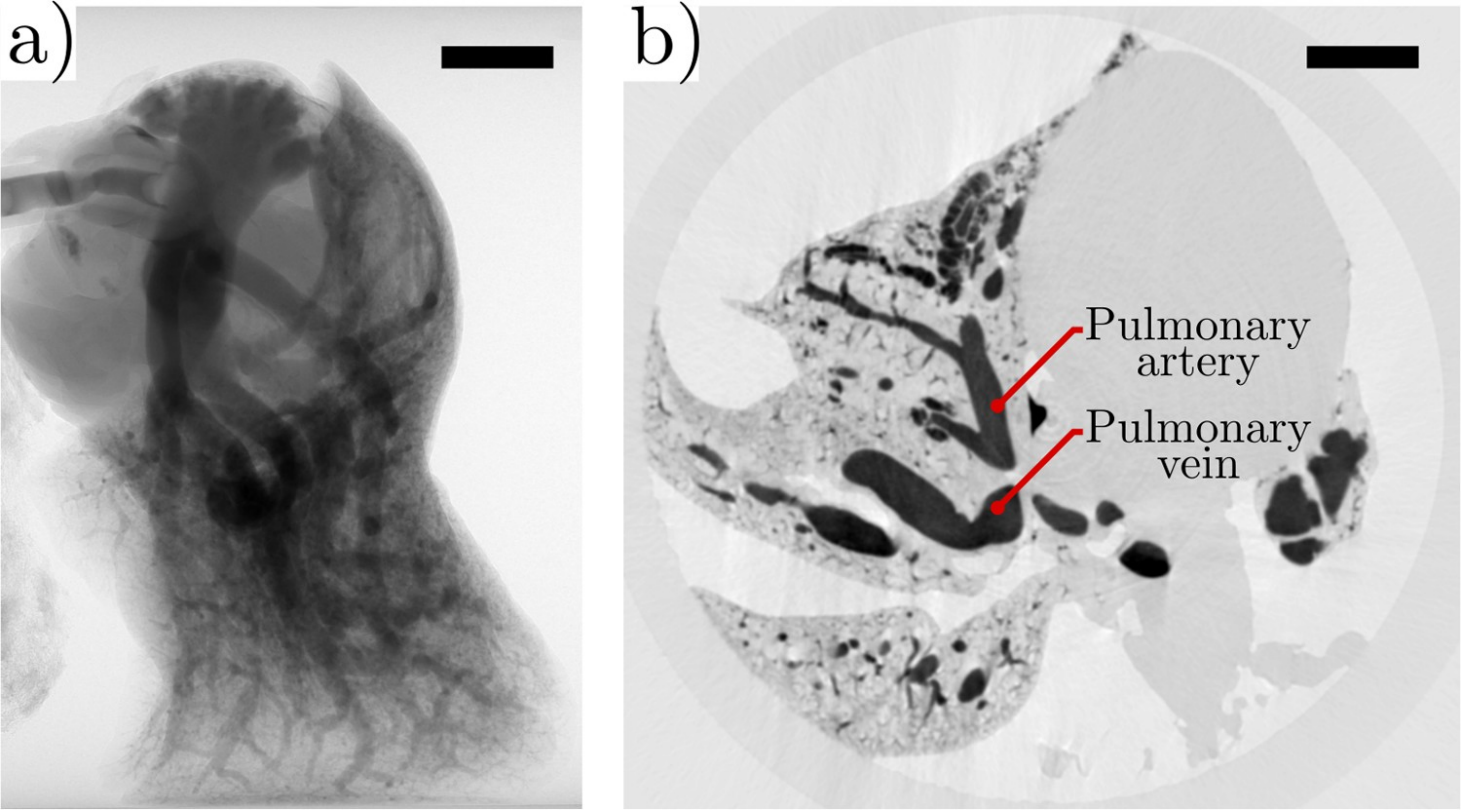
Radiography images of a whole mouse lung. a) Sample radiograph from μCT scan demonstrating successful opacification of the pulmonary circuit. b) A cross-sectional reconstructed image where darker regions correspond to higher relative local densities of contrast agent. Reconstructed slices were acquired using NRecon (Bruker Corporation). Scale bar represents 2 mm.

In this study, 16-bit grayscale images (Fig 2A) were captured using the X-ray μCT system and reconstructed using a commercial software package (NRecon, Bruker Corporation) to produce 3D images representing the vasculature of each scanned sample. Fig 2B shows a single slice of a 3D image of pulmonary vasculature. All reconstructed images were subsequently converted to 8-bit grayscale for image processing efficiency.

The reconstructed image stacks were first filtered using a Gaussian blur to reduce the intensity variance of vessels and void space, followed by a Hessian-based vesselness filter to increase the contrast between vessels and void space thereby ameliorating the segmentation process (see the Gaussian blur and Vesselness filter sections for a detailed explanation). In this study the term void space refers to any parts of the image that do not represent contrast-filled vasculature

#### Gaussian blur

Some major pulmonary arteries and pulmonary veins were clearly visible in the grayscale images (Fig 2B); however, in general, voxel intensities varied significantly within vessels with an average min/max intensity difference of 34.5% in vessels and 40.7% in void space. Min/max intensity differences were determined via random sampling of several sections of the grayscale images. These differences were assumed to be imaging artefacts or bubbles in the contrast agent (for the case of the vessels) that posed challenges to accurately distinguishing vessels from void space. To overcome these variances, a Gaussian blur [31] with a sigma value (σ) of 5 voxels was applied to the intensity of each voxel, *I*_*i*,*j*,*k*_, in the reconstructed images, where (*i*,*j*,*k*) was the location of the voxel. The Gaussian blur is a low-pass filter that is implemented by convolving the intensity of each voxel (center of the localized region) with a Gaussian kernel. A Gaussian kernel is a matrix with size equal to the localized region and populated with values reflecting a Gaussian distribution, with the peak value located at (*i*,*j*,*k*). The size of the localized region is defined by σ that corresponds to the number of voxels in each radial direction of the voxel at (*i*,*j*,*k*). The result of the Gaussian blur is a single output value, *I*_*i*,*j*,*k*_^*’*^ that replaces *I*_*i*,*j*,*k*_ from the input image.

When the Gaussian blur filter was applied to each voxel in the reconstructed images, the min/max intensity difference was reduced from 34.5% to 17.4%, and the average variance for the void space was reduced from 40.6% to 17.6%. Reducing these variances was a key step in accurately distinguishing the vessels from the void space and preventing discontinuities from manifesting within vessels during segmentation.

#### Vesselness filter

**Fig 3:**
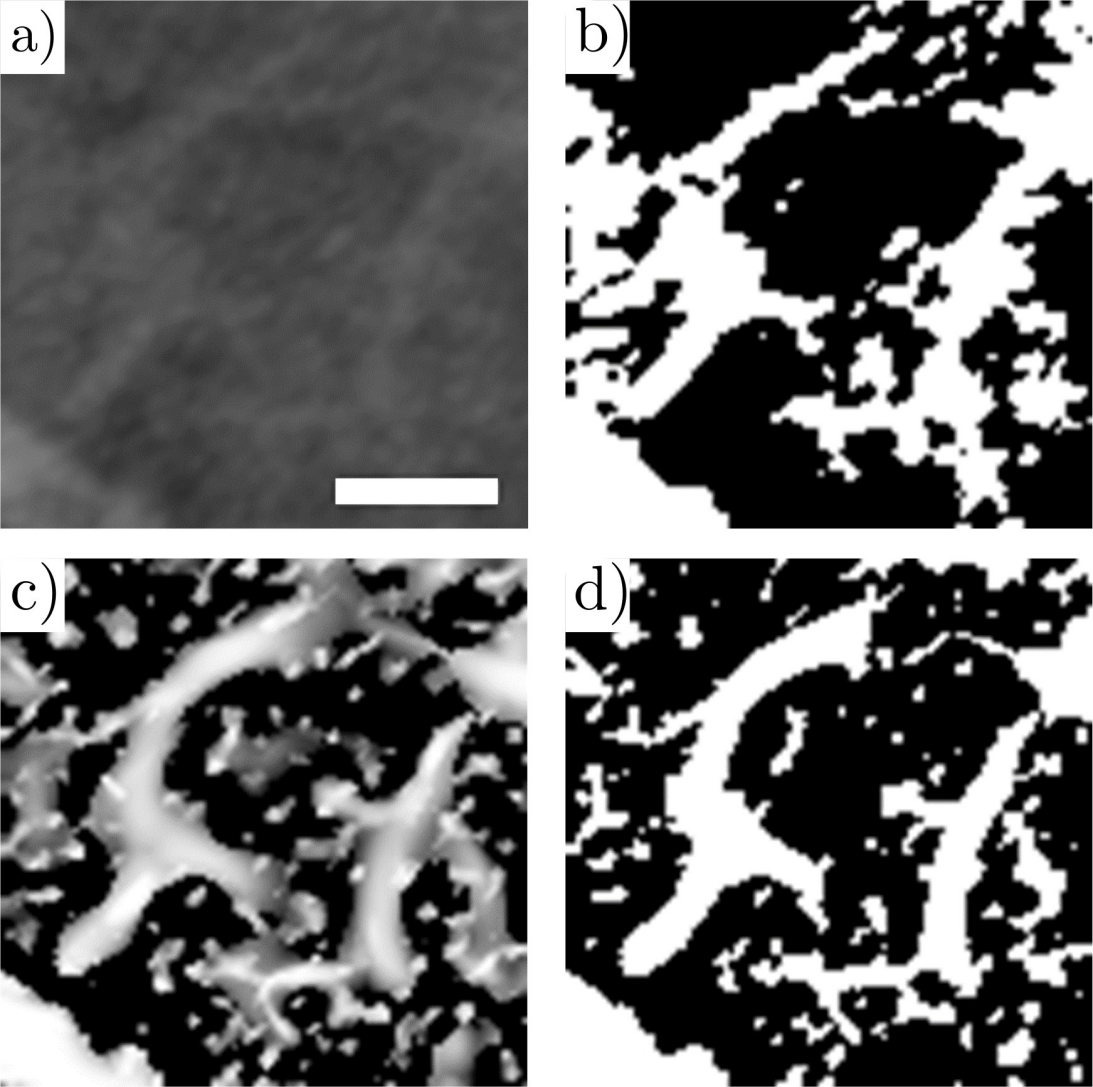
Tomographic slices of mouse lung vasculature, with cropped region shown for illustrative purposes. a) Reconstructed image slice. b) Binary image resulting from a global auto threshold using Otsu’s method on a). c) Grayscale image after applying the vesselness filter to a) with a sigma range of 1:10. d) Binary image resulting from a global auto threshold using Otsu’s method on c), after the vesselness filter was applied. Scale bar represents 150 μm and applies to all four images.

Implementing the Gaussian blur led to more distinguishable vasculature; however, the borders between vessels and void space still presented low signal-to-noise ratios (SNRs) and therefore the images were not yet suitable for accurate segmentation (binary separation between vessels and void space). After the Gaussian blur, the average SNR of the vasculature was 1.65, and for regions containing small vessels (diameter < 50 μm), such as those depicted in Fig 3A, the average SNR was 1.31. SNR values were determined by comparing vessel intensities to tissue intensities via random sampling of several sections of the grayscale images after the Guassian blur was implemented. The Hessian-based algorithm proposed by Jerman *et al*. [17] was used as a vesselness filter to enhance the contrast between vessels and void space (and thereby increase the SNR).

The vesselness filter was only applied at a σ range of 1 to 10 voxels, since the filter was not effective for enhancing vessels with a wide range of diameters. For example, when σ > 10, an artefact occurred whereby small vessels merged and manifested as non-physical large vessels. Therefore, to account for larger vessels, a second image stack (herein referred to as the *thresholded image stack*) was manually thresholded to identify vessels not detected using the selected σ range and remove vessels that were detected in the vesselness filter. A binary threshold intensity value of 115 was used to identify vessels not detected by the vesselness filter. This threshold was determined by examining gray values of vessels outside of the sizes detected by the vesselness filter from as small as 11 voxels in radius to vessels over 250 voxels in radius. The value of 115 was found to be the gray value at which the edges of these vessels were starkly visible, but the edges of smaller vessels were not. Sample images and intensity plots are available as supplementary material (S1 Fig). These vessels were preserved and labelled with the maximum vesselness probability value of 2^8-1^.

Finally, a third image stack (herein referred to as the *filtered image stack*) was generated, whereby each voxel was assigned the highest corresponding intensity when comparing the vesselness image and the thresholded image stack.

#### Segmentation

**Fig 4:**
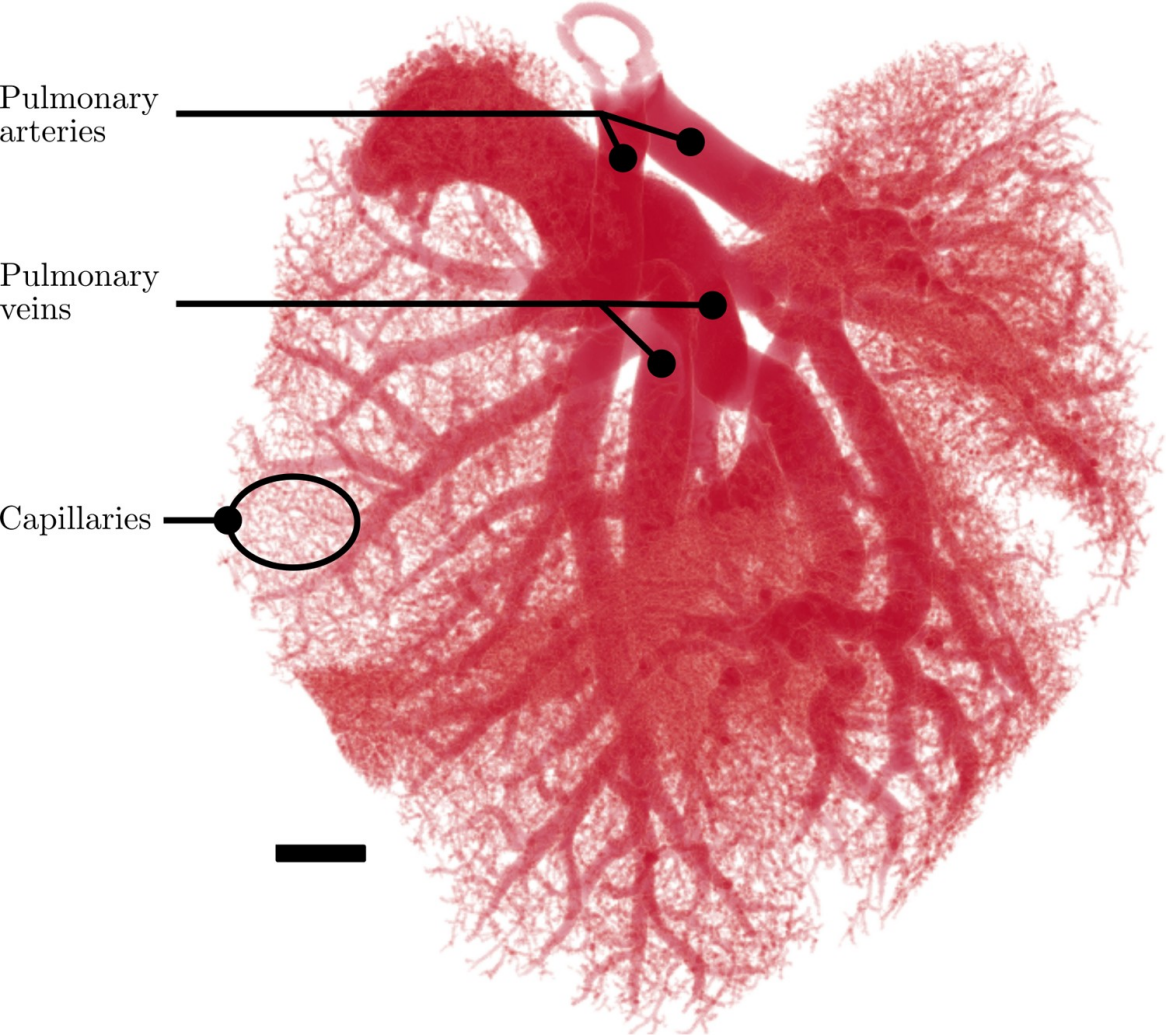
3D reconstruction of mouse lung vasculature after filtering, global auto-thresholding, and isolation of the largest voxel cluster. The branching of the pulmonary circuit is visualized. Large pulmonary arteries and veins are observed as well as micro-vasculature that make up the capillary bed. Scale bar represents 1 mm.

The first step of segmentation was to implement Otsu’s method [32] to binarize the filtered image stack and create the segmented image stack. Next, disconnected clusters of voxels in the binarized image were removed from the pulmonary circuit since we expect the vasculature to be fully connected. The largest cluster of connected voxels in the binarized image was identified and preserved whereas all other non-physical clusters were removed. These discarded clusters were removed because they did not resemble the vasculature, and we attributed their presence to the ethanol-soaked cotton that surrounded the samples that absorbed contrast agent. As an extra precaution, these discarded clusters were analyzed manually to avoid the removal of vasculature that may have been physically disconnected due to artefacts introduced by the filtering process or by contrast agent separation during injection. In Fig 3, the results of thresholding a section of the vasculature (cropped for simplicity) without applying the vesselness filter were compared to the results after applying the vesselness filter. The image in Fig 3A originally had an average SNR of 1.31; however, after applying the vesselness filter, an average SNR of 24.30 was achieved, and the complete vasculature was clearly distinguishable from void space. A 3D rendering of the segmented vasculature for the lungs is presented in Fig 4, where the branching nature of the pulmonary circuit is observed.

#### Skeletonization

Once the fully segmented vasculature was obtained from the previous image processing steps, we determined the centrelines of the vasculature as the first step towards identifying individual vessels. The centrelines of the vasculature were acquired via the skeletonization of the segmented image stack using the Skeletonize 2D/3D plugin in Fiji (ImageJ and first presented by Lee *et al*. [33]). Skeletonization was used to iteratively thin the segmented vasculature by sequentially removing 1 layer of border voxels (thickness of 1 voxel) until a series of *branching centrelines* was acquired. The resulting image is a fully connected set of voxels representing the centrelines of the segmented vasculature (herein referred to as the *vasculature skeleton*).

### Vessel network extraction

In this section, a novel Vessel Network Extraction algorithm was developed and implemented using the vasculature skeleton and segmented image stack to locate and characterize each vessel by determining its length, tortuosity, diameter, and connectivity. Each branching centerline was classified as either a *vessel centreline* (centreline of a true vessel) or a *spurious branch* (non-physical centrelines caused by noise), and the spurious branches were removed. The first implementation of a gradient-based filter, diameter-length, and FMM filter is then presented for the robust and consistent removal of spurious branches while preserving the true centerlines of the vasculature. The vessels were then characterized using the vessel centrelines and volumetric data from the segmented image stack.

#### Branching centreline and junction identification

**Fig 5:**
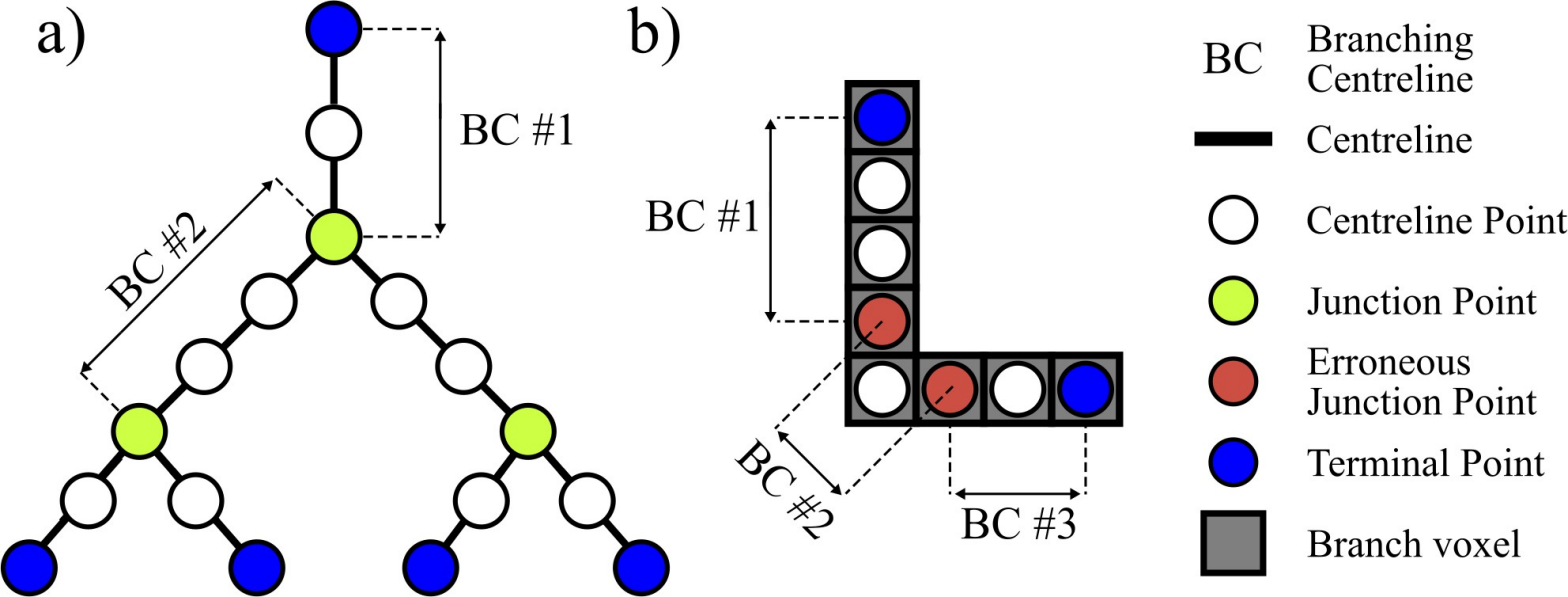
Schematic representation to illustrate defined terms used in branching centreline and junction identification. a) Definitions of centrelines, centreline points, junction points, and terminal points. Labels also show how vessels can be defined as a set of centreline points connecting a terminal point and a junction point or a set of centreline points connecting two junction points. b) Example of an L-shaped vessel where two pixels could be erroneously labelled as junction points.

The identification of junction points was a key step to identifying the coordinates of the branching centrelines. *Junctions points* were identified as voxels with three or more neighbouring voxels (Fig 5A).

Our approach to avoiding erroneous junction points is explained with the L-shaped centreline shown in Fig 5B. Specifically, the erroneous identification of three branching centrelines in Fig 5B was avoided by using the constraint that junction points must connect to at least three centreline points (not including junction points). Additionally, centrelines that looped back to the same junction point or to the same junctions as a shorter branch were considered erroneous and removed.

#### Artefact removal from skeletonized images

A significant artefact from vessel boundary noise is the manifestation of non-physical centrelines in the vasculature skeleton known as spurious branches. Although the Gaussian blur and vesselness filter reduced the prominence of this artefact by smoothing the vessel boundary, spurious branches were still abundant, particularly for large vessels (diameter > 250 μm). The most commonly reported method of eliminating spurious branch artefacts is “pruning”, which can be performed manually [20] or automatically [21]. In pruning, a threshold is applied based on the length of branches ending in a terminal point (Fig 5A) (herein referred to as *terminal branches*). Although pruning is effective, images of vasculature–such as those used in this study–with a wide range of vessel lengths (10 to 3500 μm) present a challenge because spurious branches may exhibit similar lengths to physical vessels. Specifically, when we implemented pruning as suggested in [20,21], we observed the erroneous removal of thousands of true centerlines. Therefore, we found that these past algorithms were not appropriate for our work. Rather than pruning spurious branches based on their length, we applied a *gradient filter*, a *diameter-length* filter, and finally an *FMM filter*. The filters described in this sub-section were only applied to terminal branches so as not to remove vessels essential to the connectivity of the pulmonary circuit. However, since some spurious branches had bifurcated into terminal branches, we needed to address more than one generation of spurious branches. In particular, some parent spurious branches had not been considered as terminal branches and were not eliminated after the first application of the filters. The filters were therefore reapplied to remove the spurious branches that had not been previously considered as terminal branches during the first pass of the filters. This process was repeated iteratively until all spurious branches were removed. For our samples, 7 iterations of the three filters were implemented to remove all spurious branches from the vasculature skeleton. The remaining branches were considered vessel centrelines for the remaining steps of the extraction processes. Empirical values used in this study are normalized with respect to voxel resolution and are recommended based on the samples used in this study; however future users of these methods are encouraged to assign the appropriate values for their own studies.

#### Gradient filter

In this study, we developed a gradient filter based on the description outlined by Selle *et al*. [23] to remove spurious branches from the vasculature skeleton. Prior to implementing the gradient filter, we first found the order of the voxels that comprise each terminal branch using Djikstra’s shortest path algorithm [34]. In this filter, *gradient values* were calculated for each terminal branch using the following definition:

$$Gradient=\frac{\sum_{i=1}^{n}{|\varepsilon }_{i}-\varepsilon_{i+1}|}{n*{\Delta \varepsilon }_{max}}\leq 1$$ (1)

where *ε_i_* is the distance in voxels between voxel *i* to the vessel boundary in the segmented image, and voxel *i* is a voxel in the current terminal branch. The distance *ε_i_*, was determined by applying a distance transform [35] to the segmented image stack. The number of voxels in the current terminal branch is *n*, and Δ*ε_max_* is the maximum change in distance from the vessel boundary between any voxel within the current terminal branch. The final gradient values for each terminal branch ranged from 0 to 1. A value of 0 indicates that a terminal branch followed the same trajectory as the vessel boundary because the distance to the vessel boundary remained constant. A value of 1 indicates that the terminal branch was oriented towards the vessel boundary (spurious). For an anatomical vessel, the distance from the majority of voxels in the centerline to the vessel boundary will always be less than the distance to the tip of the vessel. Thus, true terminal vessels will not be erroneously filtered by this filter as they will not demonstrate a gradient.

**Fig 6:**
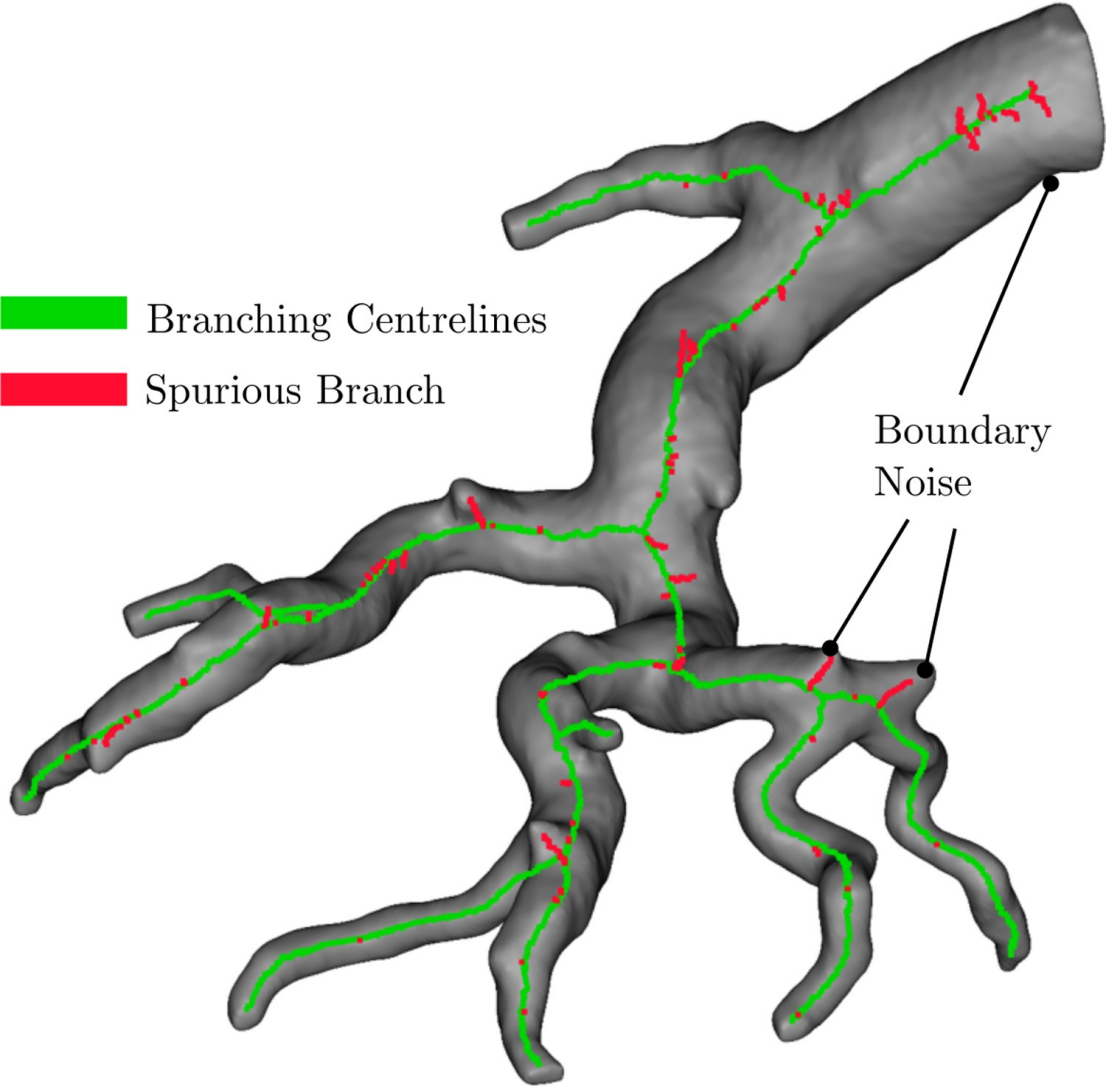
Centrelines of branching vessels highlighting removed spurious branches (red) and the final improved centreline (green).

For the image stacks in this study, a terminal branch with a gradient value greater than 0.35 was classified as a spurious branch and removed from the network. This value was assigned by qualitatively observing whether the correct branches were removed from various selected volumes of the vasculature, such as the one depicted in Figs 6 and S2. The gradient value of 0.35 in conjunction with the diameter-length and FMM filters resulted in only the removal of spurious branches and the preservation of all physical branches among the five selected volumes.

#### Diameter-length filter

After applying the gradient filter, some spurious branches were still observed. These branches remained because they did not yield sufficiently large gradient values during the implementation of the gradient filter. To identify and remove these remaining spurious branches, a diameter-length filter was created. First, diameters and lengths were pre-emptively assigned to terminal branches using the methods described in the following section. The diameter-length filter was then used to remove terminal branches that exhibited diameter-length ratios greater than a specified threshold value. The threshold was initially set to the average diameter-length ratio of arterial and venous vasculature in adult mice (~0.65) as reported by Counter et al. [4]. The threshold value was then refined to a value of 0.60 by qualitatively observing whether the correct branches were removed using sample vasculature volumes.

#### Fast marching method (FMM) filter

In this study, the FMM was used to march from each centreline voxel in the segmented image radially outward until its corresponding vessel boundary was reached. For detailed background on the FMM the reader is referred to established works in the literature [36–38]. We modified an existing FMM code (Kroon, 2001, S1 Table) to track the coordinates of the nearest centreline voxel to each voxel in the image with a non-zero intensity. Each voxel was then assigned the corresponding vessel ID of its nearest centreline. Thus, each voxel in the segmented image was assigned to a centreline and labelled as a separate cluster of voxels. From there, the outer layer of voxels (voxels bordering neighbouring clusters) were identified. The neighbourhoods of these interfacial voxels were then analyzed to determine which neighbouring clusters surrounded each cluster the most. Logically, clusters mostly surrounded by voxels with a value of zero (representing void space) were classified as belonging to true vessels. Thus, centrelines of clusters that were not mostly surrounded by voxels with a value of zero (determined by the 70th percentile) were removed.

#### Spurious branch removal validation

We employed three means of validating the effectiveness of our branch filtering and spurious branch removal. Namely, we measured significant decreases in the average variances of the diameters and lengths when we applied our method. Further, we also employed a qualitative assessment of the sample vasculatures to identify the significant improvement in branch filtering outcomes (Figs 6 and S2). Finally, we used a distance transform method to get an approximate estimation of vascular volume before and after spurious branch removal. This check resulted in a total volume estimation of 826% and 164% of the true volume of the segmented image before and after spurious branch removal, respectively. Since a distance transform finds the smallest distance to void space, it is expected that an underestimation of volume would occur if this method was used to determine diameter of each vessel. Therefore, a larger overestimation would indicate the presence of significantly more spurious branches or other noise acting as false vessels and an underestimation would indicate few or no spurious branches or other noise.

### Vessel characterization

Each vessel in the pulmonary circuit was characterized by determining its length, diameter, and connectivity by using the vessel centrelines and the volumetric information obtained from the segmented image stacks. To keep track of each vessel, the vessel centrelines were assigned unique identification (ID) numbers. First, the length of each vessel, *L* (in voxels), was determined by summing the Euclidean distance between sequentially neighbouring voxels along the centreline of each vessel, where the order of these voxels was determined via Djikstra’s shortest path algorithm [34]. The diameter of each vessel was calculated by first finding the volume of each vessel. The volume of each vessel was found via a novel implementation of the fast-marching method (FMM) [36–38], where the FMM marches from each centreline voxel in the segmented image radially outward until its corresponding vessel boundary is reached. Each voxel was then assigned the corresponding vessel ID of its nearest centreline. Finally, the volume of each vessel, *V* (in voxels), was determined as the total number of voxels assigned to each vessel centreline. The equivalent diameter, *D* (in voxels), was calculated assuming each vessel was cylindrical:

$$D=\sqrt{\frac{4V}{\pi L}}$$ (2)

**Fig 7:**
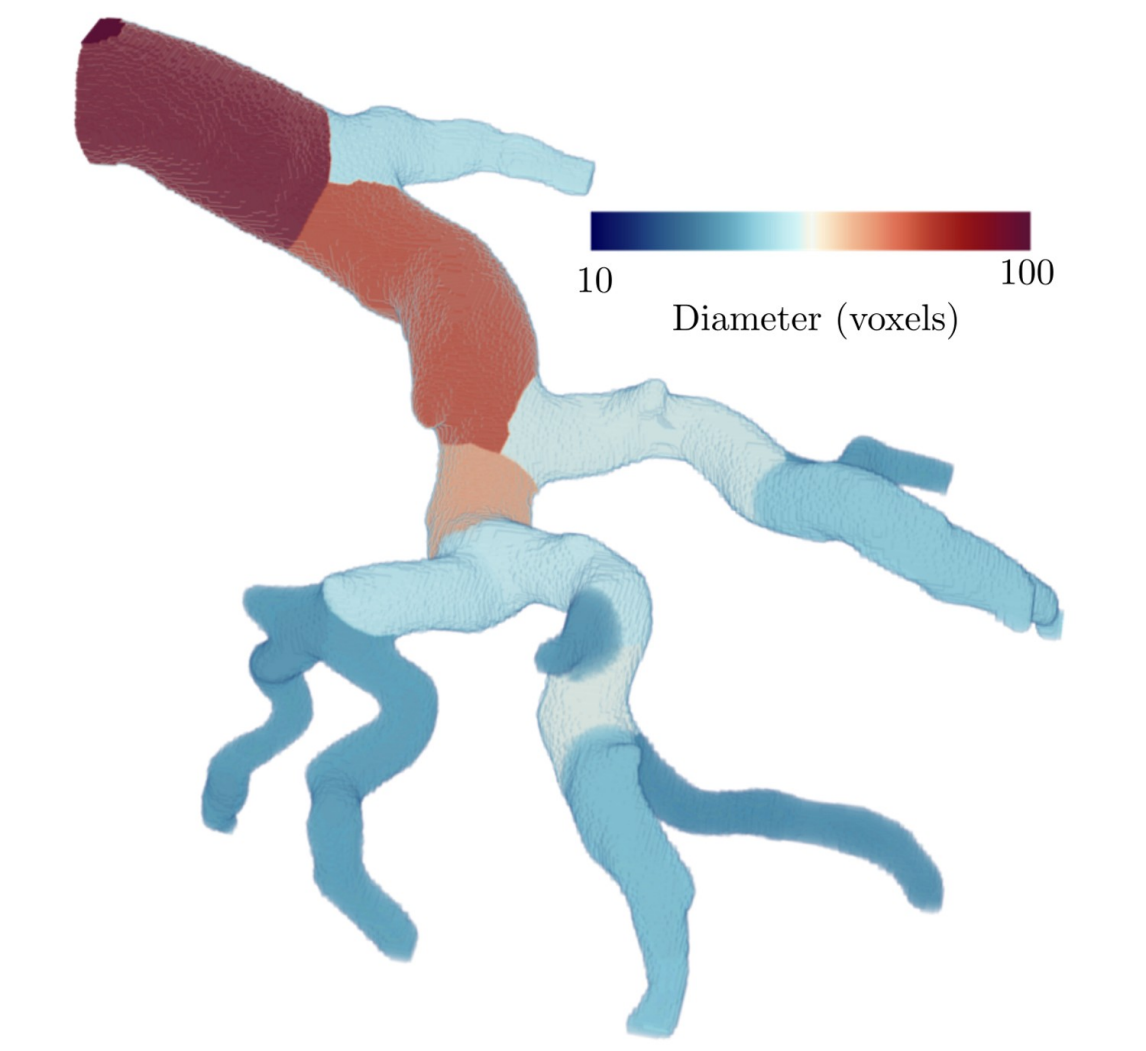
3D reconstruction of branching vessels where each voxel is coloured to represent vessel diameter. Larger vessels are darker shades of red and smaller vessels are darker shades of blue. Voxels were assigned to each vessel based on proximity to its centrelines using the fast marching method.

The diameters and lengths were converted from voxels to μm by multiplying by the voxel size of the μCT images. Fig 7 shows a visual representation of the result of the FMM algorithm used to find the diameter of vessels, where the colour of each voxel corresponds the diameter of its assigned vessel. By implementing this modified FMM to determine the coordinates of the closest centreline voxel, we avoided the erroneous assignment of a voxel to the centreline of another vessel that is nearby but not physically associated with the voxel. Furthermore, the use of a simple distance transform or a skeletonization process [23] for determining diameter led to significant underestimates. In contrast, our method led to more precise diameter approximations. Specifically, using the distance transform method on our sample vasculature images led us to achieve an average vascular volume of 76.8 *plus/minus* 0.09% of the true volume of the segmented image. Using our FMM method led to an estimated vascular volume of 100% of the true volume of the sample vasculature images.

Vessel connectivity was determined based on whether junction points were shared between vessels. For example, a vessel connected to a junction point would be connected to all other vessels connected to that junction point.

### Branching morphology characterization

**Fig 8:**
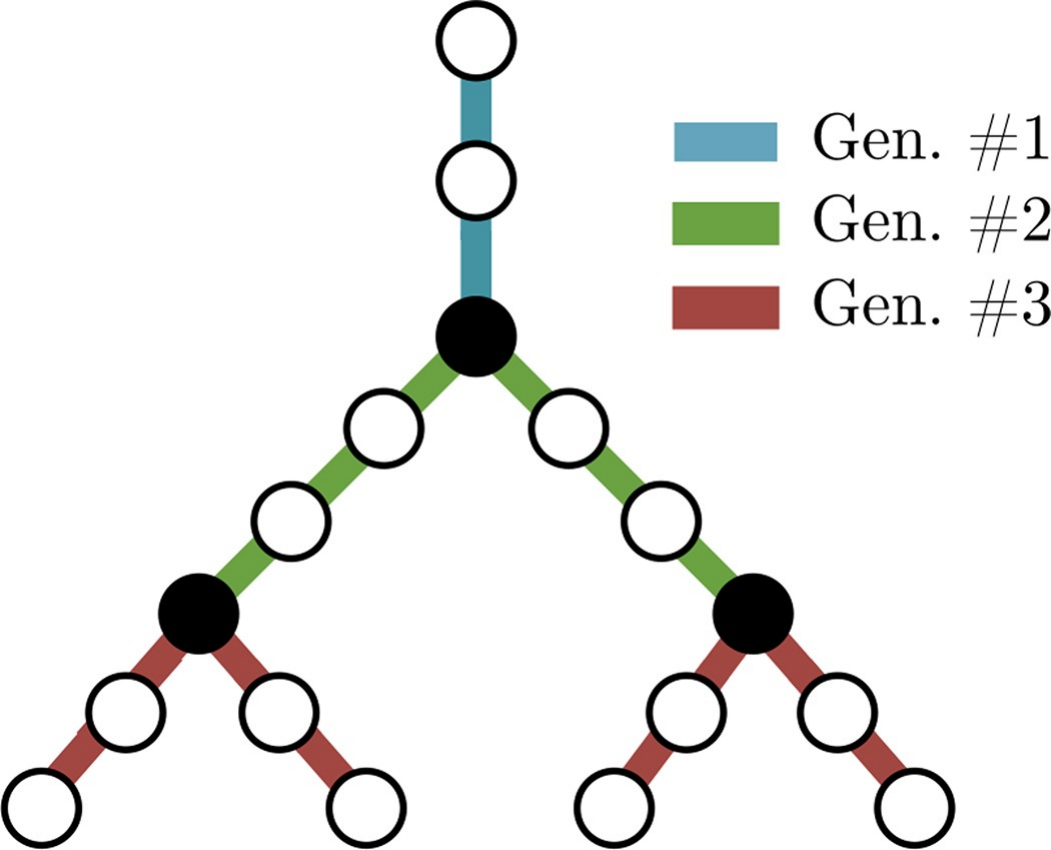
Schematic illustrating the classification of generations. Each time the vasculature reaches a junction point (solid black dots), a new generation is created including all vessels that sprout from the junction points reached by the previous generation.

To characterize the branching morphology of the pulmonary circuit, vessels were classified into generations based on their connectivity. Starting with the left and right pulmonary arteries and main pulmonary vein (identified in the segmented image manually), each time the vasculature reached a junction point, a new generation was defined which included all vessels that had branched from the junction points reached by the previous generation. Thus, generations were classified starting from the largest arterial and venous vessels and ending with the smallest terminal vessels. This process was continued until all vessel connections were accounted for (i.e. at the final generation). An example of the method used to determine the generation of each vessel based on the connectivity of the pulmonary circuit is shown in Fig 8. Once generations were determined, the total number of vessels per generation as well as the average diameter and length of each generation were determined. The collection of this information is referred to as the *generational model*.

Using the generational model, two clear ends to the pulmonary circuit were identified as the left and right pulmonary arteries and the main pulmonary vein. The main pulmonary artery was damaged too greatly by the cannulation during contrast agent injection to be identified. Pathways (series of vessels) between the arterial and venous sides of the pulmonary circuit were then identified. The mid-point of each path was identified as the vessel with the smallest diameter in the path (herein referred to as *connecting vessels*), and the vessels on either side of this vessel were classified as either part of the arterial side or the venous side. Connecting vessels are still part of the venous or arterial networks; however, in order to distinguish between the two branches of the vascular network, the classification of connecting vessels was created. A selection of these connecting vessels was further analyzed to scrutinize whether the connections were in fact connecting vessels rather than non-anatomic loops. Connecting vessels from generations 4 to 8 were analyzed qualitatively due to their likelihood of being IPAVA, since they resided in generations with larger average vessel diameters (61–172 μm). If a connecting vessel in a generation between 4 to 8 did not resemble a physical vessel, but rather two nearby vessels merging due to signal noise, it was classified as a non-anatomic loop and removed from the network. However, if the connection did resemble a physical vessel, it was classified as an IPAVA. In the remaining generations, connecting vessels were all assumed to be capillaries, since non-anatomic loops at the capillary scale are nearly indistinguishable from true connecting vessels. However, it is important to note that some neighbouring vessels within the pathways used to find connecting vessels could also be capillaries, but the methods presented here do not contain enough criteria to classify every capillary in the observable network.

## Results and discussion

In this section we first present the impact of removing spurious branches from the vasculature skeleton on the accuracy of the extraction algorithm by comparing extraction results before and after the application of the spurious branch filters. Next, we present the length, diameter, and connectivity of each vessel of the pulmonary circuit derived from our Vessel Network Extraction algorithm and compare our results to previous studies as a benchmark. Finally, we present the complete characterization of the vasculature, where the arterial and venous sections of the pulmonary circuit are distinguished. We also present the first quantitative identification of IPAVA in the pulmonary circuit via X-ray μCT.

### Impact of spurious branch filtering on pulmonary circuit morphology

**Fig 9:**
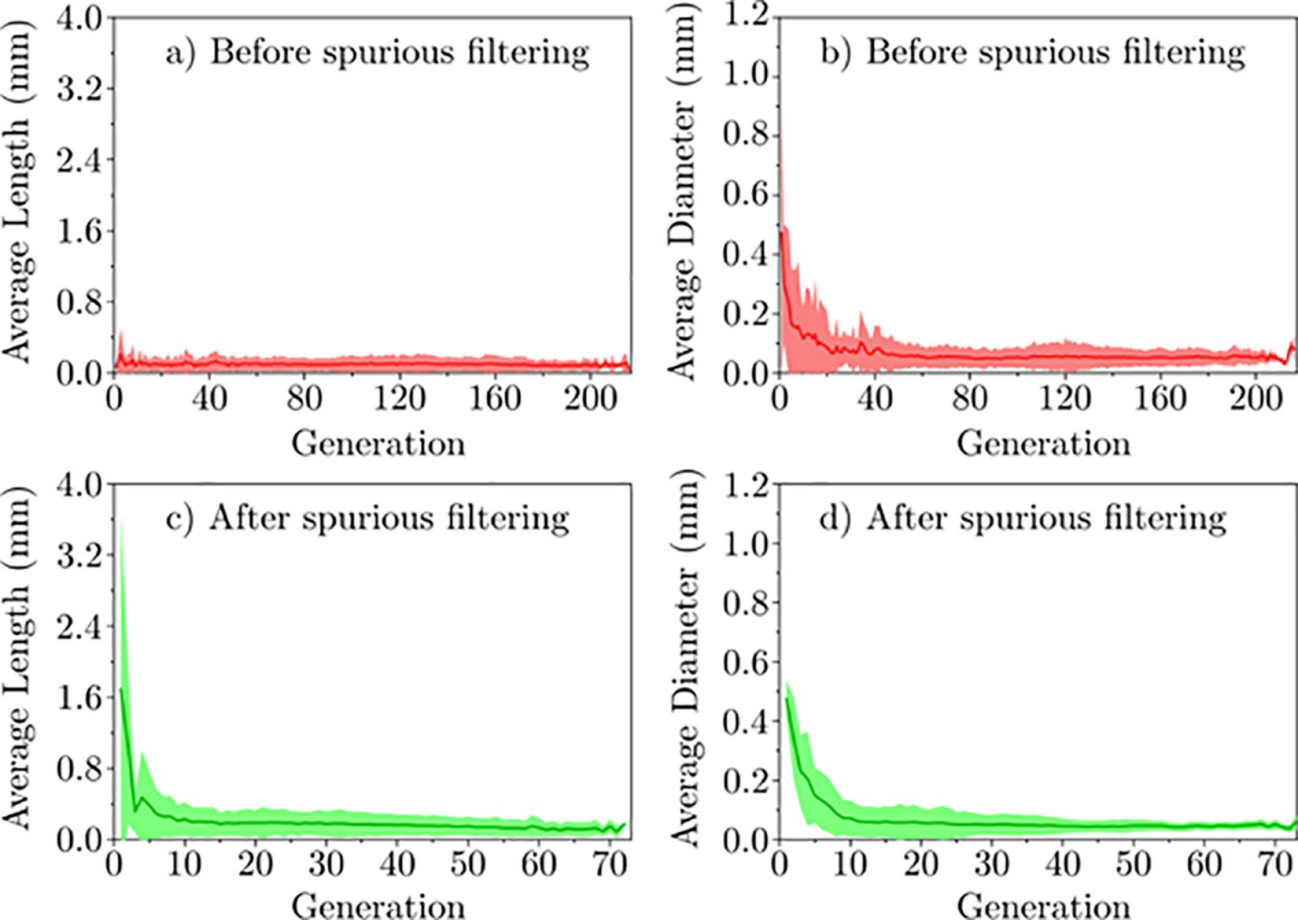
Pulmonary circuit morphology of the mouse lungs before the spurious filtering was characterized. a) average vessel length as a function of generation and b) average vessel diameter as a function of generation. Pulmonary circuit morphology after the spurious filtering was characterized: c) vessel length as a function of generation and d) vessel diameter per generation. Generations start with the left and right pulmonary arteries and main pulmonary vein. Shaded area represents one standard deviation.

Characterizing diameter and length distributions via generation number is widely adopted in the body of literature [4,11,39–43]; therefore, the following results have been reported with respect to generation number for effective information dissemination and sharing. The morphology of the pulmonary circuit before and after the application of the spurious branch filters (Fig 9) showed that spurious filtering reduced the average variance of vessel diameter (from 0.11 mm to 0.04 mm) and led to the expected exponential decay in vessel length and diameter as functions of generation before reaching a floor in vessel diameter as a result of voxel resolution limitations. The abundance of spurious branches prior to filtering was supported by the observation of shorter vessel lengths, small diameters, and a higher number of generations before spurious branch filtering due to erroneous bifurcations of the branching centrelines caused by spurious branches. The existence of erroneous bifurcations prior to spurious branch filtering provides an explanation for consistent vessel lengths across all generations in Fig 9A and 9B, in disagreement with trends of vessel lengths in previous studies [4,11]. After spurious branch filtering, a wider range of vessel lengths and fewer generations were observed.

We also expected generations to contain vessels of similar diameters in each generation due to the branching nature of the pulmonary circuit [4]. Therefore, the reduction in diameter variance demonstrated the efficacy of spurious branch filtering for removing spurious branches that would have otherwise resulted in erroneous vessel characterization. Smaller diameters in Fig 9B are attributed to fewer vessels being assigned to the volume of each vessel centreline as a result of additional centrelines (i.e. spurious branches). We observed spurious branches to be most prevalent in larger vessels (> 0.1 mm in diameter). This was also evidenced by the lower average diameter per generation. Therefore, spurious branch filtering is strongly recommended as a key step in the characterization of vasculature from 3D images (CT, MRI, etc.), particularly if the images contain large vessels relative to the spatial resolution.

### Vessel characterization

The Vessel Network Extraction algorithm was implemented in MATLAB to successfully characterize each vessel in the pulmonary circuit of the set of lungs, revealing the expected exponential decay in vessel size (calculated based on diameter and length) from the pulmonary arteries and veins to the capillaries, as shown in Fig 9C and 9D. The exponential decay pattern in vessel lengths and diameters with respect to generations is expected in branching vasculature, such as the pulmonary circuit, whereby each vessel bifurcates into smaller vessels (in length and diameter) after each junction point. In generations 111–72, the diameters and lengths became more consistent across generations, which was also expected since the higher generations are typically populated with capillaries. The exponential decay pattern and subsequent plateau in vessel lengths and diameters are in agreement with previous studies of mouse pulmonary vasculature [4,11] and other vasculature in rodent organs [39,40]. This agreement demonstrates that our in-house algorithm is able to accurately resolve the characteristics of vessels (including some capillaries) in more generations than previously attainable [4,11]. However, at the spatial resolution used (14.91 μm), it is unlikely that all capillaries were accurately resolved due to the relatively lower expected capillary size of 3–13 μm in rats (in lieu of a reliable source for mice capillaries) [41].

### Shunting evidence observed via generational model

**Fig 10:**
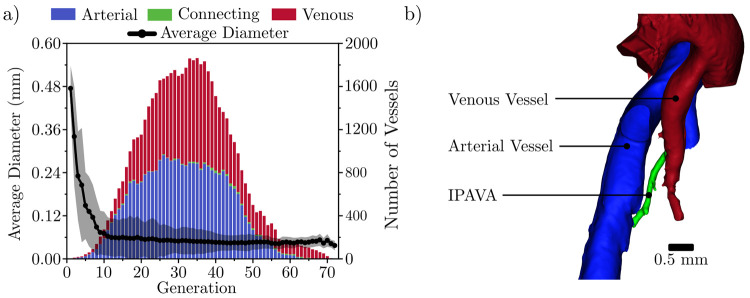
All vessels in the full lung classified as arterial, venous, or connecting vessels (including shunts). a) Average diameter of each generation in the pulmonary circuit of the mouse lungs, where the first generation includes the left and right pulmonary arteries and the main pulmonary vein. The stacked bar graph shows the total number of vessels per generation, differentiating between arterial, venous, and connecting vessels. The shaded region shown is one standard deviation of the vessel diameters. b) Example of an intrapulmonary arteriovenous anastomoses (IPAVA) (green) in the pulmonary circuit represented in a 3D model. The IPAVA connects two larger vessels belonging to the arterial and venous sides of the pulmonary circuit.

**Fig 11:**
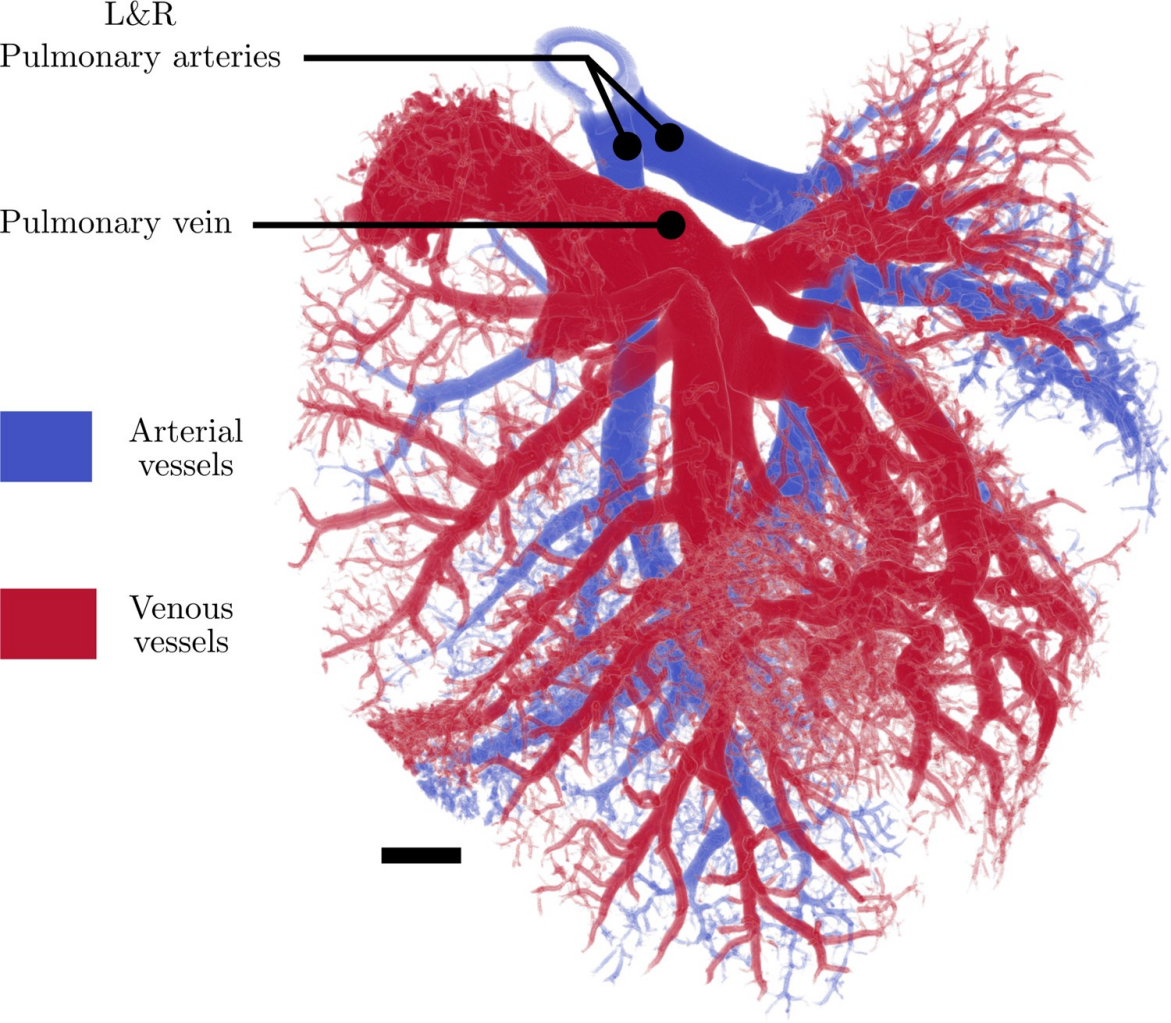
3D representation of the pulmonary circuit of the mouse lungs. Arterial (blue) and venous (red) sides of the circuit are highlighted. Scale bar represents 1 mm. Capillaries and other parts of the microvasculature were manually removed from Fig 11 in some regions so that they did not obscure the arterial vessels that appear behind the venous vessels.

The vessels in the pulmonary circuit were classified as arterial, connecting, and venous vessels to determine the branching morphology (Fig 10A). The stacked bar graph in Fig 10 shows the total number of vessels per generation in each of the aforementioned categories of vessels. The line graph shows the average vessel diameter for each generation including arterial, venous, and connecting vessels. In Fig 10, generation 1 includes only the left and right pulmonary arteries and the main pulmonary vein. In total, 72 generations were resolved in the entire network, which is considerably higher than previously reported in literature [41]. This higher number of generations is attributed to the high spatial resolution used in this study as well as the robustness of the extraction code. However, it should be noted that at this high spatial resolution, we approached but did not reach the smallest feature size expected in small rodent vasculature [41]. Therefore, the higher generations resolved in this study may still contain unidentified artefacts. Additionally, any capillaries resolved using this method were given a hierarchical generation number in the same fashion that non-capillary vessels were assigned generation numbers in this study. In reality, capillaries form a meshwork with connectivity similar to the that of links in a net. On the other hand, the larger arterial and venous branches form a hierarchy with connectivity similar to that of branches in a tree [44–46]. Therefore, the use of generations may not be as applicable for analyzing capillaries as it is for analyzing the larger branches in the network (the majority of the vessels resolved in this study). The separated arterial and venous vasculature is visualized as a 3D model (Fig 11) to demonstrate the efficacy of this separation method qualitatively.

The capillary is the most common type of connecting vessel and is where each pulmonary artery and vein meet to exchange CO_2_ for O_2_ with the alveoli. Connecting vessels were most prominent in generations 17–46, where vessels with diameters as small as 8 μm were resolved and approximately 0.28% of vessels in these generations were < 20 μm. The connecting vessels in generations 17–46 as well as the vessels < 20 μm in diameter were believed to be capillaries based on the diameter ranges of arterial vessels identified in previous studies [39]. However, at the spatial resolution used (14.91 μm), it is unlikely that all capillaries were accurately resolved due to the relatively lower expected capillary size of 3–13 μm in rats (in lieu of a reliable source for mice capillaries) [41]. For more detailed view of our reconstructed microvasculature, the reader is directed to S3 Fig.

Connecting vessels were also observed as early as generation 4 which lead to the hypothesis that IPAVA may have been detected in the vasculature. IPAVA are vessels that prematurely connect the arterial and venous trees prior to the connections in the capillary bed [26]. Previous studies [11,24,25] have typically classified these pre-capillary connections as noise acquired during the imaging phase. Therefore, further qualitative analysis was done to explore our hypothesis of IPAVA. The connecting vessels found from generations 4 to 8 exhibited diameters of 61–172 μm, which were significantly larger than the pulmonary capillary diameters and were therefore classified as potential IPAVAs. A qualitative analysis via 3D visualization was performed on the potential IPAVAs and their connecting veins and arteries. After manual inspection, the connecting vessels, such as the one shown in Fig 10B, appeared as vessels rather than non-anatomic loops and thus were classified as IPAVAs [26]. Additional angles of this IPAVA are shown in S4 Fig. In past literature, anastomoses may have been classified as noise [11,24,25], but with the new insights provided in our work, we provide a highly unique capability for researchers to identify anastomoses via the elucidation of connecting vessels in the absence of prohibitively destructive imaging that may otherwise obscure the features being imaged.

## Conclusions

In this study, we presented a new method that provides an automated characterization of the mouse pulmonary circuit via high-spatial resolution X-ray μCT imaging. Our high spatial resolution enabled us to quantify more generations than have been previously reported in literature; thus, we were able to resolve capillaries in addition to the larger vessels of the pulmonary circuit. However, additional methods are needed to distinguish all capillaries from larger arteries and veins and classify them as a meshwork rather than as a hierarchical network with generations. Our methods include a robust and detailed description of how to remove spurious branches from the skeletonized vascular image, which is adjustable to the noise level of the input images. We introduced methods to separate the venous and arterial sides of the pulmonary circuit using the connectivity of vessels. Through this method we identified vessels responsible for connecting the arterial and venous sides of the pulmonary circuit. From the connecting vessels, we further identified IPAVA. Identifying and quantifying capillaries in the pulmonary circuit via 3D imaging techniques will advance the study of diseases in the pulmonary circuit by enabling the assessment of damage due to disease at the site of gas exchange. The capability of locating and quantifying IPAVA will advance the study of vascular hypertension [14], where shunting action (via IPAVA) has been known to be triggered by hypertension [47].

This work also offers a computationally efficient foundation for modelling fluid flow in vascular systems. Our method provides a comprehensive characterization of the vasculature whereby each vessel is characterized by its length, diameter, and connectivity. Simulations based on the characterized vasculature provided here may, for example, involve modelling blood flow through vasculature [7,48,49] or flow of endothelial cells through vasculature during organ regeneration. However, for future studies involving modelling fluid flow in a vascular network extracted using our methods, researchers should be aware of the imaging limitations discussed in this study. Since the smallest blood vessels have not been resolved, we suggest that in future works, researchers should focus on portions of the vasculature of interest or apply an estimated resistance in place of the smallest vessels (i.e. the complete capillary bed) not captured in the imaging.

This work has led to an improved accuracy of lung vasculature characterization for use in a multitude of medical fields such as pathology [2–4,50,51], tissue engineering [10], and surgical planning [20,52–54]. The methods and data presented in this study can also be used to aid in more fundamental research regarding vasculature morphology throughout the body [49,55–57]. Additionally, the techniques described in this paper are not limited to even biomedical imaging. Applications that utilize 3D images of vessels or vessel-like structures can make use of this methodology for accurately quantifying the vessel features of the image such as the roots of plants for agricultural applications [18].

The authors would like to encourage the application of further validation methods for researchers aiming to apply our method to biological samples. However, we would like to acknowledge the significant challenge that conventional microscopy techniques pose. Namely, conventional microscopic approaches to vasculature such as that discussed here would only provide highly limited two-dimensional views of the vessel architecture that would be most likely compromised by the destructive nature of two-dimensional sample preparation (histology, SEM, TEM). This study serves as a demonstration of novel methods for resolving higher orders of generations and the identification of anastomoses in vasculature networks. Future studies with additional samples will provide valuable insight into the full capabilities of the proposed methods and provide metrics for the prevalence of findings such as anastomoses.

## Supporting information

S1 Table External codes used in this study. (PDF)

S1 Fig Intensity plots and corresponding grayscale images of vasculature prior to vesselness filtering and segmentation. (TIF)

S2 Fig Additional sample vasculature volume used for visual validation of the spurious branch removal methods. (TIF)

S3 Fig 3D Reconstructions of microvasculature in different regions (a) to (c) with colouring to show vessel diameter in μm. Smaller vessels are depicted as pink and purple, while larger vessels are blue and turquoise.
(TIF)

S4 Fig 3D reconstruction of an IPAVA connecting two large vessels from the arterial and venous sides of the pulmonary vasculature. Various angles (a) to (f) are shown to highlight that this IPAVA is indeed connecting the vessels rather than passing closely by.
(TIF)
