# Supplementary material for: Vessel network extraction and analysis of mouse pulmonary vasculature via X-ray micro-computed tomographic imaging
Source: PLoS Comput Biol. 2021 Apr 20;17(4):e1008930. doi: 10.1371/journal.pcbi.1008930 (PMC8594947; doi:10.1371/journal.pcbi.1008930)
Supplement: S1 Table — (PDF) [file pcbi.1008930.s001.pdf]

Name of Code	Author(s)	Link	Software, Year Created
**Jerman Enhancement Filter**	Tim Jerman, University of Ljubljana	https://www.mathworks.com/matlabcentral/fileexchange/63171-jerman-enhancement-filter	MATLAB R2015a, 2017
**Dijkstra's Shortest Path Algorithm**	Joseph Kirk	https://www.mathworks.com/matlabcentral/fileexchange/12850-dijkstra-s-shortest-path-algorithm?s_tid=prof_contriblnk	MATLAB R2006b, 2007
**Accurate Fast Marching**	Dirk-Jan Kroon, University of Twente	http://www.mathworks.se/matlabcentral/fileexchange/24531-accurate-fast-marching	MATLAB R2009a, 2011
