# Supplementary figures and images for: Vessel network extraction and analysis of mouse pulmonary vasculature via X-ray micro-computed tomographic imaging
Source: PLoS Comput Biol. 2021 Apr 20;17(4):e1008930. doi: 10.1371/journal.pcbi.1008930 (PMC8594947; doi:10.1371/journal.pcbi.1008930)

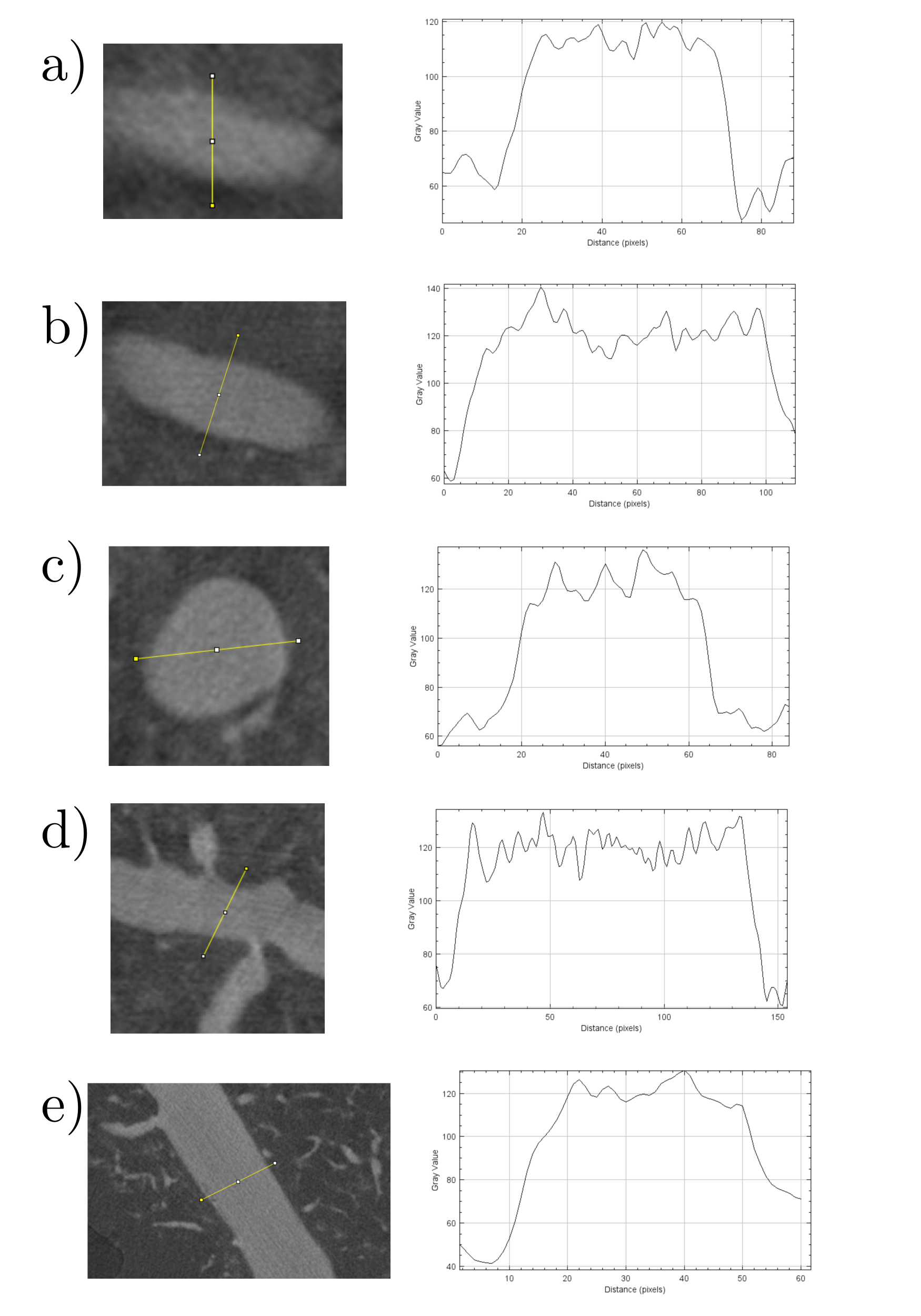

Supplement: S1 Fig — (TIF) [file pcbi.1008930.s002.tif]

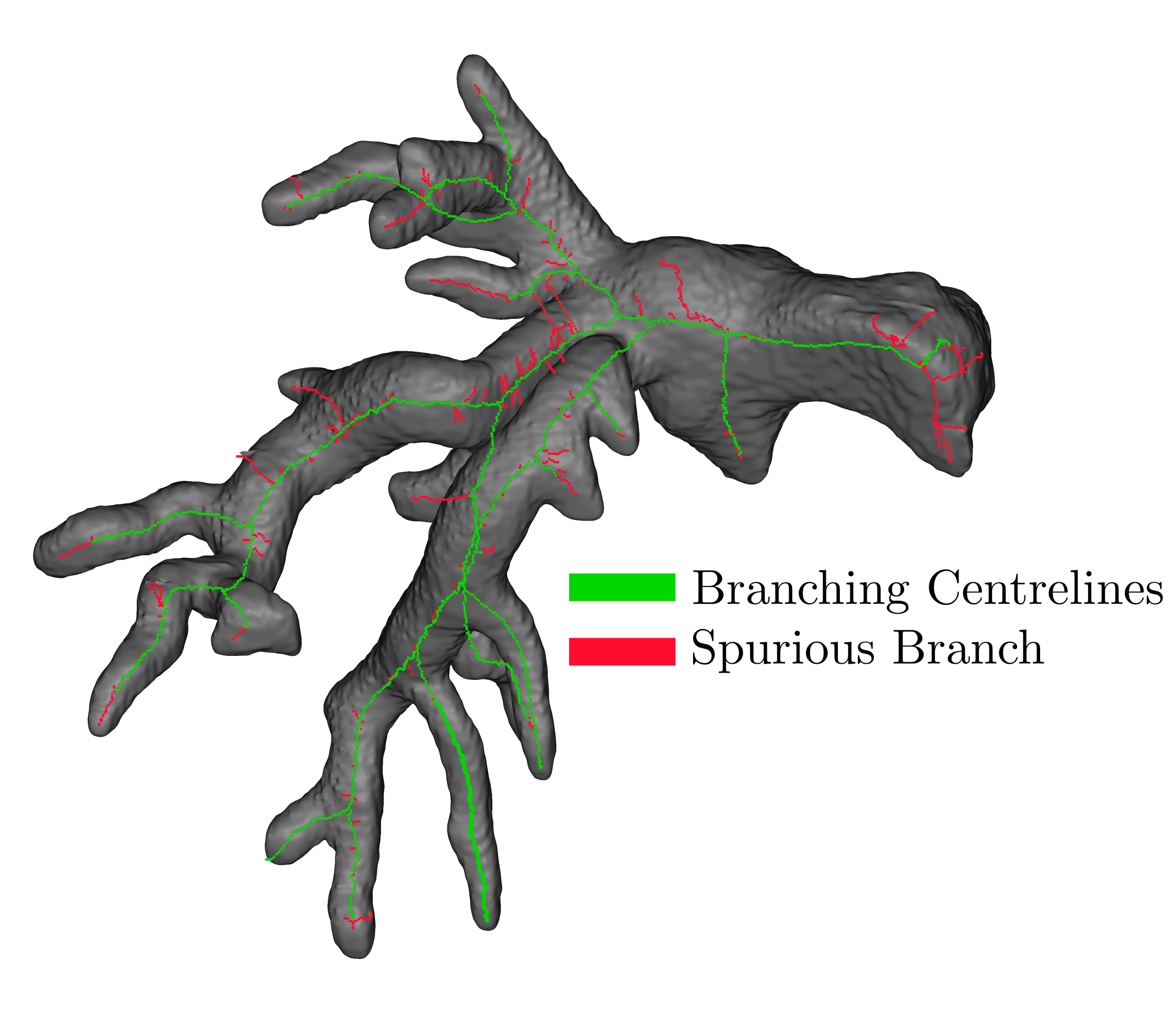

Supplement: S2 Fig — (TIF) [file pcbi.1008930.s003.tif]

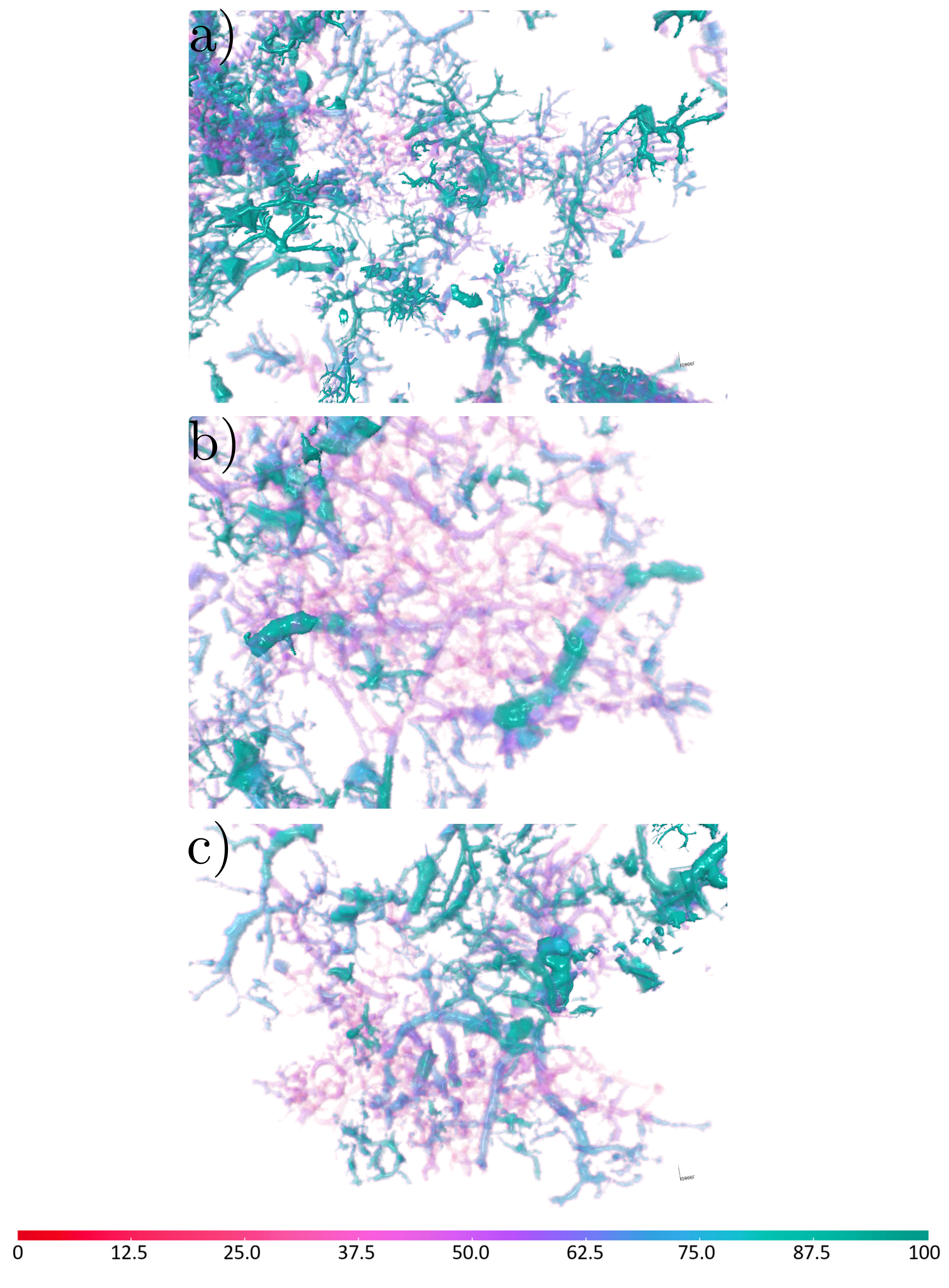

Supplement: S3 Fig — 3D Reconstructions of microvasculature in different regions (a) to (c) with colouring to show vessel diameter in μm. Smaller vessels are depicted as pink and purple, while larger vessels are blue and turquoise. (TIF) [file pcbi.1008930.s004.tif]

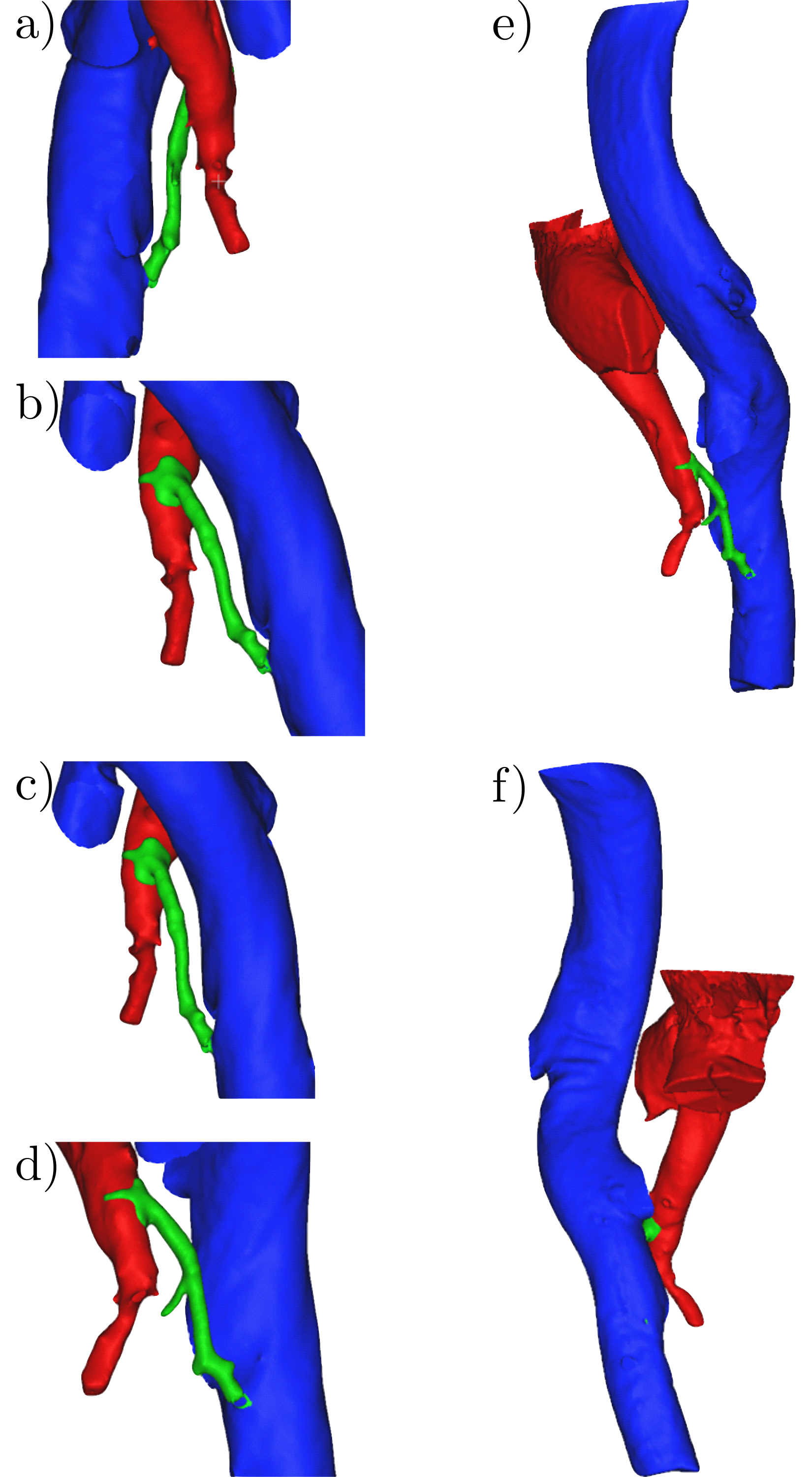

Supplement: S4 Fig — Various angles (a) to (f) are shown to highlight that this IPAVA is indeed connecting the vessels rather than passing closely by. (TIF) [file pcbi.1008930.s005.tif]
